# Tracing *Staphylococcus capitis* and *Staphylococcus epidermidis* strains causing septicemia in extremely preterm infants to the skin, mouth, and gut microbiota

**DOI:** 10.1128/aem.00980-24

**Published:** 2024-12-18

**Authors:** Forough L. Nowrouzian, Kirth Lumingkit, Monica Gio-Batta, Daniel Jaén-Luchoro, Thordur Thordarson, Anders Elfvin, Agnes E. Wold, Ingegerd Adlerberth

**Affiliations:** 1 Institute of Biomedicine, Department of Infectious Diseases,The Sahlgrenska Academy, University of Gothenburg 580950 , Gothenburg, Sweden; 2 Department of Clinical Microbiology, Sahlgrenska University Hospital 56749 , Gothenburg, Sweden; 3 Institute of Clinical Science, Department of Pediatrics, Sahlgrenska Academy, University of Gothenburg 205657 , Gothenburg, Sweden; 4 Department of Pediatrics, Sahlgrenska University Hospital, The Queen Silvia Children's Hospital 89599 , Gothenburg, Sweden; Centers for Disease Control and Prevention, Atlanta, Georgia, USA

**Keywords:** coagulase-negative staphylococci, *S. capitis*, *S. epidermidis*, extremely preterm infants, septicemia, skin microbiota, oral microbiota, gut microbiota, CoNS

## Abstract

Coagulase-negative staphylococci (CoNS) comprise about 50 species, some of which cause septicemia in preterm neonates. CoNS establish early on the skin and in the oral and gut microbiota, from where they may spread to the bloodstream. The colonization pattern preceding septicemia is not well-defined. Forty-two extremely preterm neonates (≤28 + 0 gestational weeks) were followed from birth to 2 months with regular sampling and culturing of the skin and oral and gut microbiota. Blood samples were drawn upon clinical suspicion of septicemia and cultured. CoNS species were identified using matrix-assisted laser-desorption ionization time of flight mass spectrometry (MALDI-TOF). Random amplified polymorphic DNA was used for strain typing, and strains were characterized regarding biofilm production and virulence gene carriage. CoNS blood isolates underwent whole genome sequencing. *Staphylococcus epidermidis* represented 72% of the CoNS isolates on skin or mucous membranes, followed by *Staphylococcus capitis* (13%) and *Staphylococcus haemolyticus* (7%). CoNS septicemia was diagnosed in nine infants, yielding 11 septicemia isolates: seven *S*. *capitis* and four *S*. *epidermidis,* of which nine were further analyzed. The *S. capitis* septicemia isolates belonged to the NRCS-A clone. Two-thirds of the septicemia strains were traced back to the commensal microbiota. Colonization of the oral cavity by *S. capitis* was significantly associated with CoNS septicemia development, although the blood-borne *S. capitis* strains were more commonly found on the skin than in the mouth prior to invasion. Biofilm production was not associated with septicemia. Our results implicate CoNS colonization as a step that precedes septicemia in preterm neonates. Early colonization of the oral cavity by *S. capitis* may represent a particular risk.

**IMPORTANCE:**

Septicemia is a major cause of morbidity in preterm infants. Coagulase-negative staphylococci (CoNS) can colonize skin, oral cavity, and intestines and are a common cause of septicemia in this group. The relation between CoNS colonization pattern at the species and strain level and septicemia has scarcely been studied. We mapped colonization of the skin, oral cavity, and intestines by CoNS species in extremely preterm infants and speciated and strain-typed the skin, mucosal, and blood isolates. Two-thirds of the CoNS septicemia blood strains, including a majority of *S. capitis* strains belonging to the NRCS-A clone, were tracked to the commensal microbiota. We demonstrated that CoNS species differ in their colonization patterns, whereby *S. capitis* was primarily a skin colonizer. However, its colonization of the oral cavity was enhanced among infants developing septicemia. Our study provides a starting point for further explorations of the relationship between CoNS colonization and septicemia in preterm infants.

## INTRODUCTION

More than one in four extremely preterm infants (i.e., those born before 28 full weeks of gestation) develop septicemia, which contributes to high morbidity and mortality rates among these infants (1, 2). Coagulase-negative staphylococci (CoNS) are the most common cause of septicemia in this vulnerable population (3).

However, diagnosis of CoNS septicemia is challenging, since CoNS in blood cultures may also result from contamination by commensal skin bacteria during sampling (4). In order to facilitate diagnosis, increased levels of CRP (5) or IL-6, and/or specific symptoms are commonly applied to identify CoNS-related septicemic episodes (6).

CoNS comprise some 50 different species, among which *Staphylococcus epidermidis* dominates in the skin microbiota and is also a common cause of CoNS septicemia in preterm infants (7). *Staphylococcus capitis*, *Staphylococcus haemolyticus,* and *Staphylococcus warneri* are also common skin colonizers and causes of septicemia in this group (7).

CoNS colonize not only the skin but also the oral cavity and the gut (8, 9), especially in newborns (8, 10). However, CoNS colonization of the gut and oral cavity have been poorly studied, and the impact of such colonization on the development of CoNS septicemia in preterm infants is largely unknown.

CoNS colonizing the skin may reach the bloodstream via catheters or other breaches of the skin barrier (9). However, it is also likely that CoNS in the oral cavity or gut invade the bloodstream through translocation, that is, the passage of live bacteria across the mucosal barrier (11–13), especially in preterm newborns, who have an immature immune system and poorly developed barrier functions (14). Thus, colonization at these sites may precede spread to the bloodstream.

CoNS generally are of low virulence, although certain traits have been linked to their capacities to cause infections. These traits include adhesins, which promote bacterial attachment, and the formation of biofilm (15), which protect the bacteria against antimicrobial peptides and phagocytes (16). For *S. epidermidis,* an essential factor for biofilm formation is the polysaccharide intercellular adhesin (PIA), which is encoded by the *icaADBC* operon (17, 18). In addition, accumulation-associated protein (Aap) mediates PIA-independent biofilm formation (19).

Homolog of the *icaADBC* operon has been identified in *S. capitis* (20, 21). This species also carries the *pis* gene, which encodes a plasmin-sensitive protein that shares domain and amino acid sequence homologies with Aap (22).

It is clear that certain clones or lineages of *S. epidermidis* or *S. capitis* are more apt to cause septicemia than others, for instance, *S. epidermidis*. ST2 is a leading lineage with worldwide spread that frequently carries staphylococcal cassette chromosome mec (SCCmec), insertion sequence element (IS256), and *icaADBC* operon and produces biofilm (23). Another lineage frequently associated with CoNS septicemia is *S. epidermidis* ST5 (24). Both are frequent causes of septicemia in preterm infants (25). Regarding *S. capitis*, one particular clone, NRCS-A, has been isolated from neonatal intensive care units globally. It is a frequent cause of septicemia in preterm neonates and shows resistance toward betalactams and aminoglycosides and also variable sensitivity to vancomycin, which could facilitate its dissemination in neonatal intensive care units (NICUs) where vancomycin is frequently used (26, 27).

In the present study, we describe the colonization patterns of different CoNS species on the skin, in the oral cavity and the gut of 42 extremely preterm neonates during their first 2 months of life. All isolates of *S. epidermidis, S. capitis,* and *S. haemolyticus* that originated from infants yielding at least one blood culture that was positive for CoNS were typed to the strain level, and the blood isolates were subjected to whole genome sequencing. The associations between CoNS colonization patterns and CoNS septicemia development were investigated, and causative strains were traced back to the commensal microbiota of affected infants. Finally, the carriage of putative virulence genes and biofilm production were compared between species and within species between blood-borne strains and strains colonizing skin or mucosal sites.

## MATERIALS AND METHODS

### Subjects

Forty-two extremely preterm infants were included in the present study. They were part of the ÖFLORA birth cohort consisting of 89 neonates born at ≤28 full weeks of gestation and admitted directly after birth to the NICU at the Queen Silvia Children’s Hospital in Göteborg, Sweden, between March 2013 and June 2015.

Skin, oral, and rectal/fecal samples were collected at 1 and 3–4 days of life and at 1, 2, 3, 4, 6, and 8 weeks of age. Although samples from all 89 infants were frozen for later analysis by DNA-based methods, fresh samples from 42 of the infants were analyzed directly using culture-based methods, representing the cohort included in the present study. The decision to culture samples from an infant was taken when laboratory resources were deemed to be sufficient to permit bacterial culturing and identification during the weeks following the birth of that infant. The 42 infants whose samples were cultured did not differ significantly from the 47 additional infants in the cohort with respect to any of the measured variables, except that there was a higher proportion of girls (Table 1). The number of samples collected from these 42 infants at the consecutive sampling occasions were 39, 35, 37, 35, 30, 27, and 20, respectively. Blood cultures were drawn on clinical suspicion of septicaemia.

**TABLE 1:** Characteristics of the ÖFLORA birth-cohort, with data for the infants included and the infants not included in the present study shown separately^*a*^

	Included (*N* = 42)	Not included (*N* = 47)	*P*-value
Girls	26 (62)	16 (34)	0.01
Latency period			
0–12 h	30 (71)	28 (60)	0.50
12–24 h	0 (0)	4 (9)	0.12
> 24 h	12 (29)	12 (26)	1.00
Cesarean delivery	20 (48)25 + 3 (22 + 6–27 + 5)	21 (45)25 + 1 (22 + 5–27 + 6)	0.83
Gestational age at birth (weeks)	25 + ^3^ (22 + ^6^–27 + ^5^)	25 + ^1^ (22 + ^5^–27 + ^6^)	0.32
Birth-weight, (g)	830 (415–1,235)	750 (420-1,350)	0.34
Antenatal steroids to mother^*b*^	38 (91)	37 (79)	0.24
Antibiotics to mother^*c*^	28 (67)	36 (77)	0.24
Cord blood pH	7.3 (6.8–7.5)	7.3 (6.9–7.5)	0.13
Apgar 10 min	8.0 (1–10)	8.0 (3–10)	0.38
CPAP (days)^*d*^	23 (0–72)	26 (0–76)	0.21
Postnatal steroids (days)	0 (0–28)	1 (0–42)	0.16
No. of blood transfusions	7 (0–28)	12 (0–35)	0.11
No. of plasma transfusions	3 (0–14)	3 (0–19)	0.84
Parenteral nutrition (days)	14 (1–39)	19 (4–96)	0.06
Parenteral fish oil^*e*^	13 (18)	21 (30)	0.20

^*a*^ The sub-cohort in the present study consisted of the 42 extremely preterm infants from whom fresh samples of the skin and oral and gut microbiota were analyzed by culturing, whereas samples from 47 infants in the cohort were frozen for later analysis by DNA-based methods. Data are expressed as median (range) for continuous variables or number of infants (%) for proportions. Included and non-included infants were compared using the Mann-Whitney *U*-test for continuous variables and Fisher exact tests for nominal variables.

^*b*^ Administration of two doses of betamethasone between 24 h and 7 days before delivery.

^*c*^ Administration of antibiotics to the mother ≤24 h before delivery; penicillin or cefotaxime (and in case of allergy, clindamycin) was administered according to local guidelines regarding chorioamnionitis.

^*d*^ Treatment with continuous positive airway pressure.

^*e*^ Seventy-one of the infants in the cohort (80%) were also enrolled in the Donna Mega randomized clinical trial to investigate the effect of parenteral fish oil (SMOFlipid, with 15% fish oil) rather than standard parenteral emulsion (Clinoleic) on retinopathy of prematurity (59). Data are N (%) of infants who received the parenteral emulsion containing a fish oil fraction rather than the standard parenteral emulsion, as a result of their inclusion in the trial.

The study was approved by the Ethics Committee of the University of Gothenburg (ÖFLORA 319–12 and T325-13), and informed written consent was obtained from the parents of the infants.

### Diagnostic criteria for CoNS septicemia

An episode of CoNS septicemia was defined as (i) a blood culture positive for CoNS, (ii) an increase in CRP or IL-6 from baseline, or (iii) at least one of the following signs and symptoms as a change from baseline: (i) an episode of apnea (ii), an episode of bradycardia (heart rate <100/min), or (iii) temperature <36.5° or >38°C. Furthermore, the following criteria had to be fulfilled: appropriate antibiotic treatments for >120 h and a central catheter (umbilical catheter, peripherally inserted central catheter, or a central venous catheter) in place within 48 h before the culture was drawn (6).

### Antibiotic treatment

All the infants received prophylactic treatment with penicillin G and tobramycin directly upon admission to the NICU, and this was often followed by cloxacillin treatment. More than half of the infants received vancomycin and/or meropenem for shorter or longer periods after the first days of life due to suspected or confirmed infection. The highest percentages of infants treated with these latter antibiotics were 2 weeks old (Table S1). Antibiotic treatments did not differ significantly between the sub-cohort studied here and the other infants in the ÖFLORA cohort (data not shown).

### Sampling, culturing, and species identification of CoNS

For skin samples, a swab (ESwab system; Copan Diagnostics Inc., Murrieta, CA, USA) was rolled over the skin close to the umbilical stump. For oral samples, a swab was rolled over the mucosal surfaces of the inner cheeks and tongue. For sampling of the gut microbiota, freshly voided feces were collected from a diaper and placed in a sterile tube, which was placed in an airtight bag filled with an anaerobic atmosphere (AnaeroGen Compact; Oxoid Ltd., Basingstoke, UK). When an infant did not pass any stools on a scheduled sampling occasion, a swab was inserted into the anus and rolled over the rectal mucosa. All samples were handled and cultured within 24 h after collection.

The rectal, oral, and skin swabs were shaken in their tubes (Copan transport medium) for 5–10 s (Supermixer: Lab-Line Instruments Inc., Melrose Park, IL, USA). The swabs were then discarded, and the tubes were centrifuged at 12,000 × *g* for 10 min. Most of the supernatant fluid was discarded, and the pellet was mixed with the remaining liquid (400 µL for rectal samples and 300 µL for oral and skin samples) and diluted serially in sterile peptone water. For stool samples, a calibrated spoonful of feces was serially diluted in 1 mL of sterile peptone water. Dilutions of the fecal or rectal swab samples were cultured aerobically at 37°C for 2 days on Staphylococcus agar plates (Staphylococcus Medium No 110, Oxoid CM0145) produced at the substrate unit of the Clinical Microbiology Laboratory, Sahlgrenska university Hospital.

From appropriate dilutions, free-lying colonies with different morphologies were isolated, subcultured, and confirmed to be staphylococci by typical Gram-stain appearance and a positive catalase reaction. Isolates that were negative in the coagulase test were deﬁned as CoNS (8). The limits of detection were 13 CFU/swab for the oral and skin samples, 16 CFU/swab for the rectal samples, and 316 CFU/g for the fecal samples. Matrix-assisted laser-desorption ionization time of flight mass spectrometry (MALDI-TOF) (VITEK MS; Biomerieux, Marcy l'Etoile, France) was used for species identification of the CoNS isolates.

CoNS blood culture isolates were identified to the species level using MALDI-TOF (VITEK MS), and their antibiotic susceptibility profiles were determined at the Clinical Microbiology Laboratory, Sahlgrenska University Hospital as part of the clinical routine. The isolates were stored frozen at −70°C until further analysis.

### Clone/strain analysis by random amplified polymorphic DNA (RAPD)

In RAPD, bacterial DNA is amplified using random primers under low stringency conditions, thereby creating a unique “DNA fingerprint” for each strain/clone (28). We have previously applied RAPD for strain typing of *Escherichia coli* (29) and *Staphylococcus aureus* (30). Here, we optimized the method for strain typing of *S. epidermidis, S. capitis,* and *S. haemolyticus* isolates. All the CoNS isolates from 12 infants with blood cultures that were positive for CoNS were analyzed by RAPD. In brief, 1–2 bacterial colonies cultured on tryptic soy agar (TSA) were suspended in 50 µL of 1× Tris EDTA and incubated at 95°C for 10 min. The mixture was centrifuged for 5 min at 12,000 × *g,* and the supernatant was used as the template DNA. PCR was performed using 2 µL of template DNA, 12.5 µL of HotStarTaq Master Mix (Qiagen, Spånga, Sweden), 1.8 µM of the primer 5′-AACGGTGACC-3′ (OPE-20, Kit E; Operon Technologies Inc., Alameda, CA, USA), and 3.5 mM MgCl_2_ in a final volume of 25 µL, using the following amplification program: 95°C for 15 min (heat activation), followed by 35 cycles of 94°C for 70 s, 33°C for 60 s, and extension at 72°C for 130 s, with termination at 72°C for 130 s. RAPD amplicons were separated and visualized using an automated system (Agilent 2200 TapeStation System; Agilent Technologies Inc., Santa Clara, CA, USA). Two isolates were considered to belong to the same strain if their RAPD profiles showed similarity regarding all major bands and variation in no more than 2–3 minor bands. If two isolates showed similar RAPD patterns but differed in more than 2–3 minor bands, the RAPD analysis was repeated to increase the certainty of the classification. All isolates originating from an infant were analyzed in the same RAPD run. RAPD patterns were not compared between infants.

### Assessment of biofilm formation by *S. epidermidis*, *S. capitis,* and *S. haemolyticus*

*S. epidermidis, S. capitis,* and *S. haemolyticus* strains were assessed for biofilm production using the phenotypic “tube method” (15), with some modifications. In brief, a small aliquot of bacteria from an overnight culture was inoculated into a sterile glass tube that contained 2 mL of tryptic soy broth and incubated at 37°C overnight. The broth was discarded, and the bacterial cells adhering to the tube were rinsed with phosphate-buffered saline (pH 7.3) followed by sterile water, and stained with safranin (0.1%). A negative control (*E. coli* strain HB 101, OR:K12) (31) and a positive control strain (*S. epidermidis* CCUG 31568; Culture Collection University of Gothenburg, Gothenburg, Sweden) were included in each experiment. Biofilm was considered to be present if a bacterial film lined the bottom and the wall of the tube. The amount of biofilm was semi-quantitatively assessed in relation to the positive control (strong biofilm production) and scored as absent (0), weak (1), moderate (2) or strong (3). All the strains were tested in duplicate.

### Screening for potential virulence determinants

#### Identification of the *ica* operon in *S. epidermidis* and *S. capitis*

In *S. epidermidis*, the *ica* operon was identified as described previously (32) for the amplification of *icaA, icaD,* and *icaB,* with some modifications. The primers used were as follows: icaADB-F 1893-TTATCAATGCCGCAGTTGTC-1913 and icaADB-R 2388-GTTTAACGCGAGTGCGCTAT-2408 (32). PCR was performed using 2 µL of template DNA (see above), 0.4 µM of the *icaADB-F*/*icaADB-R* primer (32), 12.5 µL of Multiplex Master Mix (Qiagen), and 5 µL Q-Solution (Qiagen) in a final volume of 25 µL, and the mixtures were subjected to initial denaturation at 95°C for 15 min, followed by 30 cycles of amplification (denaturation at 94°C for 30 s, annealing at 55°C for 60 s, and extension at 72°C for 60 s), with a final extension step of 72°C for 10 min. The PCR products were separated by agarose gel electrophoresis and stained with ethidium bromide (29). The *S. epidermidis* strains CCUG 31568 and CCUG 15605 were used as positive and negative controls, respectively.

To ensure that the *icaADB-F/icaADB-R* primer detected the entire *ica* operon, an additional PCR for detecting the *icaC* gene (also part of the *ica* operon) (33) was performed in parallel with a random subset of 30 of the 53 strains analyzed above. PCR was performed as described above, but with a higher annealing temperature of 58°C. As the methods yielded similar results, only the method of Eftekhar et al. (32) was used to detect the *ica* operon in the remaining strains.

For *S. capitis,* the *ica* operon was detected using the primers (icaAD: icaAD-F gcgccttcaattctaaaatctcccc, icaAD-R gcgcacgacctttcttaattttttgg and icaBC: icaBC-F gcgcttagtgtgatttccaactagg, icaBC-R gcgcaagaaagaaaggtggctatgctac) described previously (34)

PCR reaction was performed as described above. The PCR program was started by initial denaturation at 95°C for 15 min, followed by 30 cycles of amplification (denaturation at 94°C for 30 s, annealing at 65°C for 70 s, and extension at 72°C for 70 s), with a final extension step of 72°C for 10 min.

### Whole genome sequencing

#### DNA extraction and sequencing

All CoNS blood isolates available for analysis (*n* = 16) were subjected to whole genome sequencing. Genome sequences are available under the accession numbers included in the Table S4. Strains were grown on blood agar at 37°C for 24 h. Biomass was collected and homogenized in ATL buffer (Qiagen lysis buffer) with an enzyme mix containing lysozyme (100 mg/mL), lysostaphin (0.5 mg/mL), and mutanolysin (1 U). Samples were incubated at 37°C for 1 h and vortexed twice during this incubation period. The mixture was then transferred to a 2 mL tube containing glass beads and bead-beaten in a TissueLyser II (Qiagen) at a frequency of 25 Hz for 5 min. Next, 25 µL of Proteinase K (25 mg/mL) was added, and the samples were incubated at 56°C for 1 h. Following this, the samples were transferred (without beads) to a new 2 mL tube, and DNA was extracted using the EZ2 robot (Qiagen). Some samples required an additional DNA cleanup step using the Clean and Concentrator DNA kit (Zymo Research).

Whole-genome sequencing of the strains was carried out using Illumina technology through Eurofins Genomics (Germany). DNA libraries were prepared according to an optimized protocol with standard Illumina adapter sequences. Sequencing was performed with paired-end reads on the Illumina NovaSeq platform (NovaSeq PE 150 mode). Raw illumina sequences were assembled using SPAdes v1.13 (35). Contigs shorter than 500 nucleotides were discarded. Genome quality was assessed using the Quality ASsessment Tool (36) and CheckM (37). Taxonomic assignment of the genomes was analyzed using average nucleotide identity by BLAST, via the online tool JSpeciesWS (38). Genome sequences of the type strains *S. epidermidis* JCM 2414^T^ (BAZV00000000.1), *S. capitis* NCTC 11045^T^ (CABEEX000000000.1), *S. haemolyticus* ATCC 29970^T^ (GCF_006094395.1), and *S. aureus* DSM 20231^T^ (NZ_CP104478.1) were used as references.

#### Genome analysis

Identification of *S. capitis* strains belonging to the NRCS-A clone was performed with single nucleotide polymorphism (SNP) and clustering analysis using the Reference sequence Alignment based Phylogeny builder (REALPHY) (39) and the same reference sequences indicated elsewhere (26).

Antibiotic resistance gene identification and resistance pattern prediction were performed using ResFinder v4.6.0 (40, 41), hosted at the Center of Genomic Epidemiology platform (https://www.genomicepidemiology.org/). *In silico* PCRs for detecting the genes *icaADCB* and I*S256* were performed using FastPCR (42). Identification of SCCmec genes was done using SCCmecFinder (43).

Finally, multilocus sequence typing was conducted using the public databases for molecular typing and microbial genome diversity pubMLST (41).

### Statistical analyses

Proportions were compared using Fisher’s exact test. The median biofilm score was compared between different groups of strains using the Mann-Whitney *U*-test (SPSS Statistics ver. 25.0; IBM Inc., Armonk, NY, USA).

## RESULTS

Forty-two extremely preterm infants, born at 22^+6^–27^+5^ weeks of gestation, were included in the current study. Their birth weights were in the range of 400–1,200 g, and 20/42 infants were delivered by cesarean section (Table 1).

### Blood cultures containing CoNS and CoNS septicemia episodes

Blood cultures were drawn on clinical suspicion of septicemia. On 17 occasions in 14 infants, CoNS were isolated from the blood cultures. On 10 of these occasions, the affected infants (*N* = 9) were diagnosed as having CoNS septicemia, whereas on seven occasions, the affected infants (*N* = 7) did not fulfill the diagnostic criteria for septicemia, and the CoNS blood isolates were regarded as blood culture contaminants. The antibiotic susceptibility profiles of the CoNS blood culture isolates were determined by the Clinical bacteriology laboratory as part of routine blood culture analysis (Table S2).

### CoNS colonization at different body sites

The skin and oral and gut microbiota were sampled at regular intervals from 1 day to 8 weeks of age. All of the infants harbored CoNS on the skin, in the oral cavity, and/or in the gut on at least some sampling occasions during the study period, with the most-frequently isolated species being *S. epidermidis* (72% of all isolates), *S. capitis* (13%), and *S. haemolyticus* (7%). Less commonly detected were *Staphylococcus hominis* (3%), *S. warneri* (2%), and *Staphylococcus lugdunensis* (2%). *Staphylococcus pasteuri, Staphylococcus saprophyticus,* and *Staphylococcus equorum* were occasionally isolated (collectively, 0.5% of the isolates) (Table 2).

**TABLE 2:** Species distribution among CoNS isolates obtained from different body sites of extremely preterm infants during their first 2 months of life^*a*^

	No. (%) of CoNS isolates			
	Skin	Oral cavity	Rectum/feces	Total
*S. epidermidis*	294(64)^*c*^	317 (76)	318 (78)	929 (72)
*S. capitis*	105 (23)^*d*^	37 (9)	31 (8)	173 (13)
*S. haemolyticus*	24 (5)	31 (7)	32 (8)	87 (7)
*S. hominis*	12 (3)	15 (4)	12 (3)	39 (3)
*S. warneri*	11 (2)	9 (2)	6 (2)	26 (2)
*S. lugdunensis*	10 (2)	7 (2)	5 (1)	22 (2)
Others^*b*^	2 (0.4)	2 (0.5)	2 (0.5)	6 (0.5)
Total CoNS	458 (100)	418 (100)	406 (100)	1282 (100)

^*a*^ Skin, oral, and rectal swab samples and fecal samples were cultured for staphylococci at 1, 3–5, and 7 days and at 2, 3, 4, 5, 6, and 8 weeks of age. CoNS isolates were speciated using MALDI-TOF analysis.

^*b*^ *S. pasteuri* (n = 4), *S. saprophyticus* (n = 1), and *S. equorum* (n = 1).

^*c*^ *P* ≤ 0.0001 (lower than among isolates from other body sites, Fisher´s Exact Test).

^*d*^ *P* ≤ 0.0001, (higher than among isolates from other body sites).

The species distribution differed significantly between isolates from different body sites. *S. epidermidis* was less-dominant in skin isolates (64%) than in the oral (76%) and rectal/fecal (78%) isolates, whereas *S. capitis* was more prevalent in skin isolates (23%) than in the oral (9%) and rectal/fecal (8%) isolates (Table 2).

**Fig 1:**
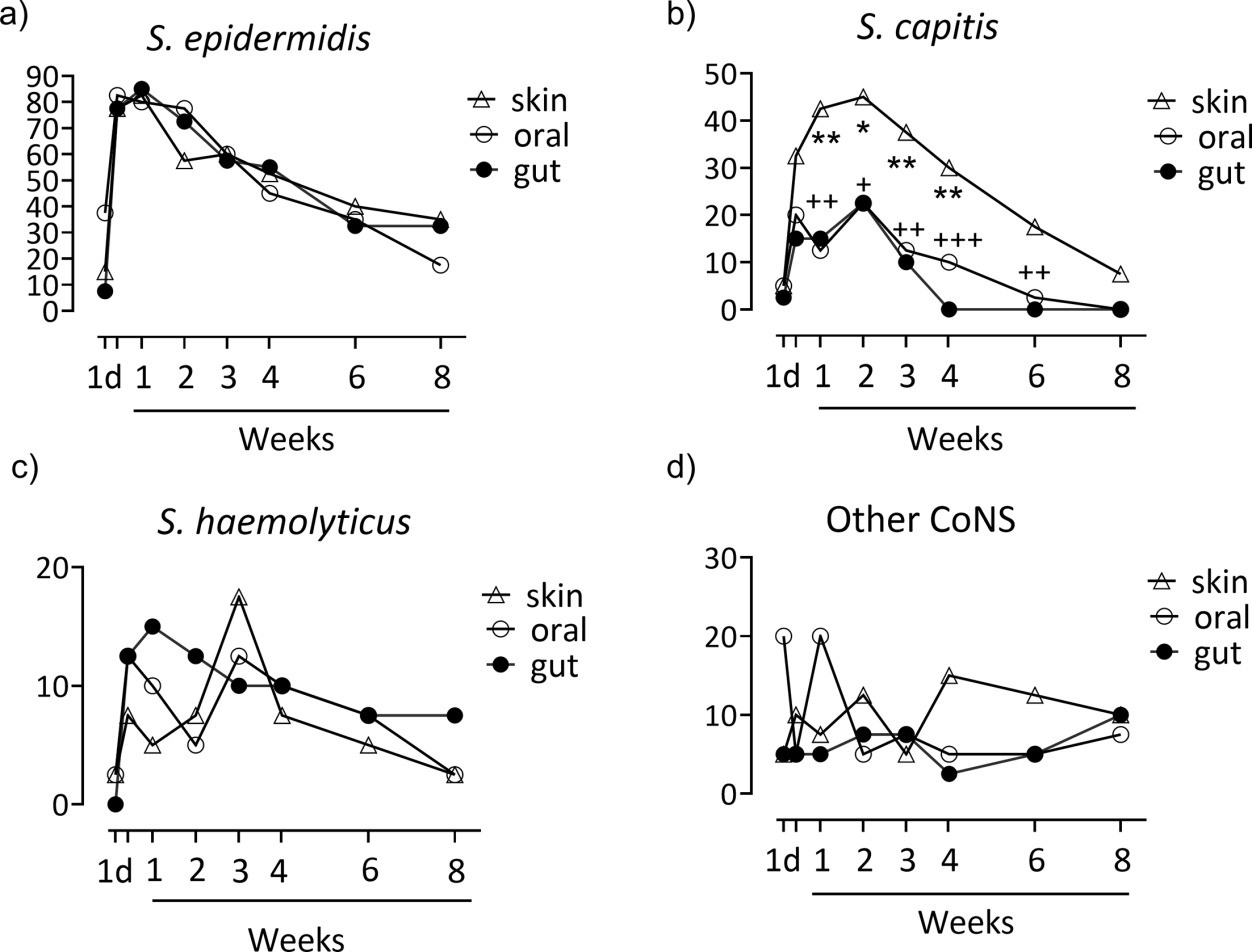
Longitudinal colonization patterns of the skin, oral cavity, and gut of 42 extremely preterm infants who were followed from 1 day to 2 months of age, shown separately for (a) *S. epidermidis*; (b) *S. capitis*; (c) *S. haemolyticus*; and (d) other CoNS species (*S. hominis*, *S. warneri*, *S. lugdunensis*, *S. pasteuri, S. saprophyticus,* and *S. equorum*; totaling 7.2% of all the isolates). For each species, the colonization rates at different body sites on each sampling occasion were compared using Fisher exact tests. A significantly higher colonization rate of *S. capitis* on the skin than in the oral cavity is indicated as: *P < 0.05, **P < 0.01, and ***P < 0.001. A significantly higher proportion of *S. capitis* on the skin than in the gut microbiota is presented as: +*P* < 0.05, ++*P* < 0.01, and +++*P* < 0.001.

The colonization patterns over time are shown in Fig. 1. *S. epidermidis* was the most-prevalent species, being isolated from the oral cavity in 38% of the infants already on the first day of life, and also from the skin (15%) and gut (7.5%) of some infants (Fig. 1a). Colonization of the oral cavity, gut, and skin increased and peaked at 1 week of age, at which point ~80% of the infants were colonized at all the investigated body sites. Colonization declined steadily thereafter (Fig. 1a).

*S. capitis* was more prevalent on the skin than in the mucosal sites (Fig. 1b). Colonization peaked at 2 weeks of age, when almost half of the infants harbored this species in the skin microbiota, and one in four was colonized in the mouth and/or gut (Fig. 1b). Thereafter, colonization declined over time at all body sites, although *S. capitis* remained more common on the skin than in the oral cavity (between 1 and 4 weeks of age) or in the gut (between 1 and 6 weeks of age) (Fig. 1b).

Around 10% of the infants were colonized by *S. haemolyticus*, with a slightly decreasing frequency over time (Fig. 1c).

The colonization rates of other CoNS species (*S. hominis*, *S. warneri*, *S. lugdunensis*, *S. pasteuri, S. saprophyticus,* and *S. equorum,* combined) did not seem to vary over time or differ between body sites (Fig. 1d).

Next, we analyzed whether the patterns of colonization by *S. epidermidis, S. capitis,* or *S. haemolyticus* over time were related to the sex of the infant, mode of delivery, gestational age at birth (≥26 weeks or <26 weeks), exposure to antibiotics during delivery, or non-prophylactic antibiotic treatment (in the period preceding sampling). Only minor differences in relation to these factors were observed. Most notable was somewhat increased skin or oral colonization by *S. epidermidis* in infants exposed to antibiotics during partus (Fig. S1a and b). The relationship between the degree of prematurity or delivery mode and CoNS colonization was negligible (Fig. S1c through f). The sex of the infants had no significant effects on CoNS colonization at any timepoint (data not shown).

We also analyzed whether treatment with specific antibiotics (cloxacillin, meropenem, or vancomycin) was related to the CoNS colonization pattern. An infant was considered to be treated if they had received the antibiotic in the sampling interval preceding the sampling occasion analyzed. The effects were small but treatment with meropenem was associated with increased early colonization of the oral cavity by *S. capitis* (Fig. S2a), but with reduced gut colonization by *S. epidermidis* (Fig. S2d). Vancomycin seemed to have no effect on *S. capitis* but suppressed gut colonization by *S. epidermidis* (Fig. S2e and f). Treatment of infants with cloxacillin did not seem to influence colonization by *S. epidermidis* or *S. capitis* (data not shown).

Due to the limited sample size, we did not adjust for potential confounders in the statistical analyses regarding associations between sex, perinatal factors, or antibiotics and the CoNS colonization pattern.

### CoNS septicemia in relation to CoNS colonization pattern

Overall, 9/42 (21%) infants included in the current study developed CoNS septicemia at least once during their first 2 months of life. The septicemia episodes yielded 11 invasive CoNS isolates (causing septicemia). An additional five infants (12%) had blood cultures that were positive for CoNS (eight isolates), although these were all deemed to be the result of blood culture contamination (Table S3).

**Fig 2:**
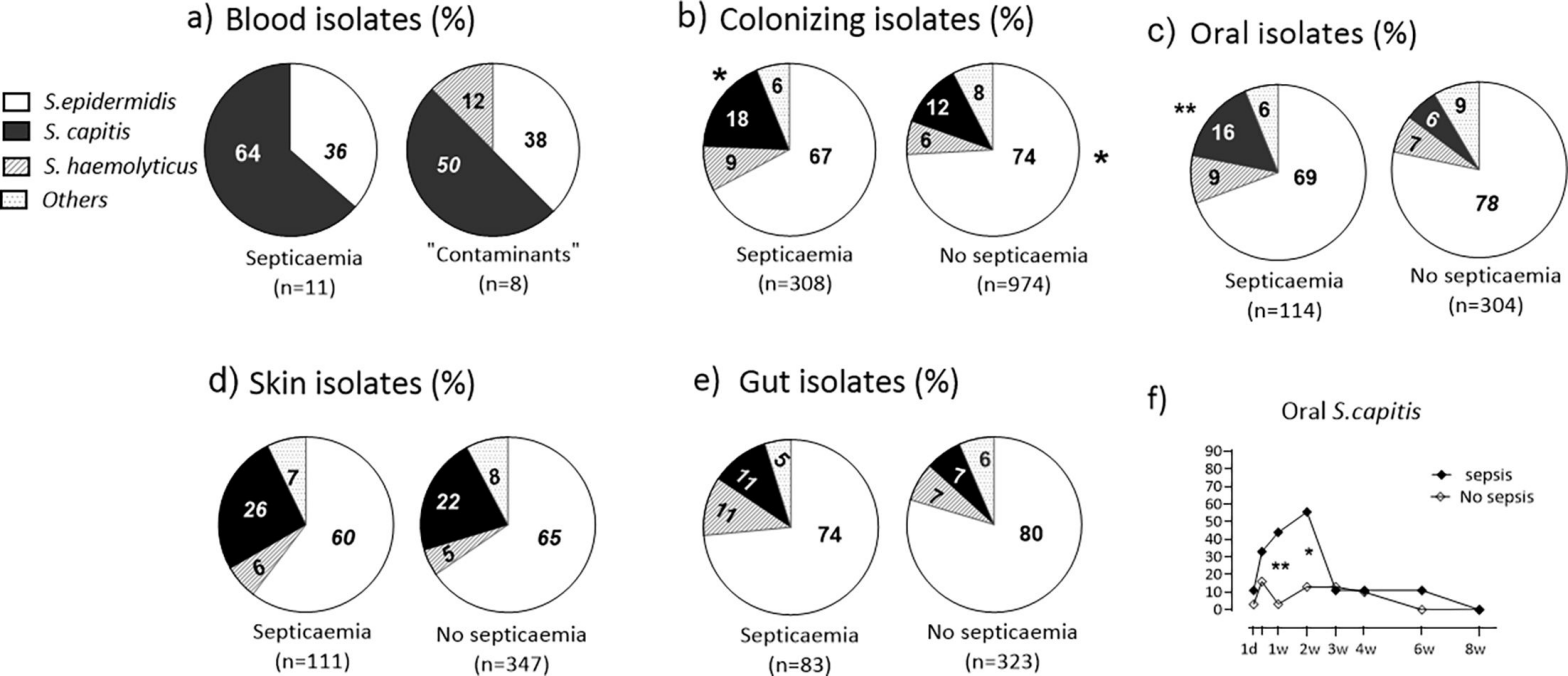
Species distributions among (a) CoNS septicemia blood isolates (*n* = 11) and CoNS blood isolates regarded as blood culture contaminants (*n* = 8). (**b-f**) Comparison of species distributions among colonizing CoNS isolates from any site (b), the oral cavity (c), the skin (d), or rectum/feces (e) of the nine infants who developed CoNS septicemia and the 33 infants who did not. (f) Rate of colonization by *S. capitis* of the oral cavity in infants who did (*N* = 9) or did not (*N* = 33) develop CoNS septicemia during the first 2 months of life. Proportions were compared using Fisher exact tests. *P < 0.05 and **P ≤ 0.01.

*S. capitis* was the most common species among the CoNS septicemia isolates (7/11, 64%) and also among the blood culture contaminants (4/8, 50%), followed by *S. epidermidis* (4/11, 36% of septicemia isolates; 3/8, 38% of blood culture contaminants) (Fig. 2a). The proportions of these species in the commensal microbiota (skin, oral cavity, and gut isolates) of the nine children with CoNS septicemia and infants with no CoNS septicemia (*N* = 33) are shown in Fig. 2b. *S*. *capitis* was significantly enriched among the septicemia blood CoNS isolates (64%), compared with commensal isolates (18%) from infants who were developing septicemia (*P* = 0.001). In contrast, *S. epidermidis* was less frequent among septicemia blood isolates than among commensal isolates from the corresponding infants (36% vs 67%, *P* = 0.05).

Furthermore, we compared the CoNS species distributions in the commensal microbiota (skin, oral cavity, and gut isolates) between infants who developed or did not develop septicemia (Fig. 2b). *S. capitis* constituted a higher proportion of the commensal isolates from infants who developed septicemia than from those who did not (18% vs 12%, *P* = 0.01, Fisher exact test), whereas *S. epidermidis* constituted a larger share of the commensal isolates from infants who did not develop septicemia (74% vs 67%, *P* = 0.02).

Regarding colonization by CoNS at different body sites, infants who developed CoNS septicemia had a significantly increased proportion of *S. capitis* among oral isolates compared with other infants (16% vs 6%, *P* = 0.003) (Fig. 2c), whereas no significant differences were observed regarding the proportions of *S. capitis* among gut or skin isolates in infants with or without CoNS septicemia (Fig. 2d and e).

As shown in Fig. 2f, oral colonization by *S. capitis* was significantly increased at 1 week of age (44 vs 3.2%, *P* = 0.006) and 2 weeks of age (55 vs 13%, *P* = 0.01) in infants who developed septicemia (from any type of CoNS), but not at later sampling occasions (Fig. 2f).

### Strain typing of *S. epidermidis*, *S. capitis,* and *S. haemolyticus* blood and commensal isolates in infants who had positive blood cultures

**Fig 3:**
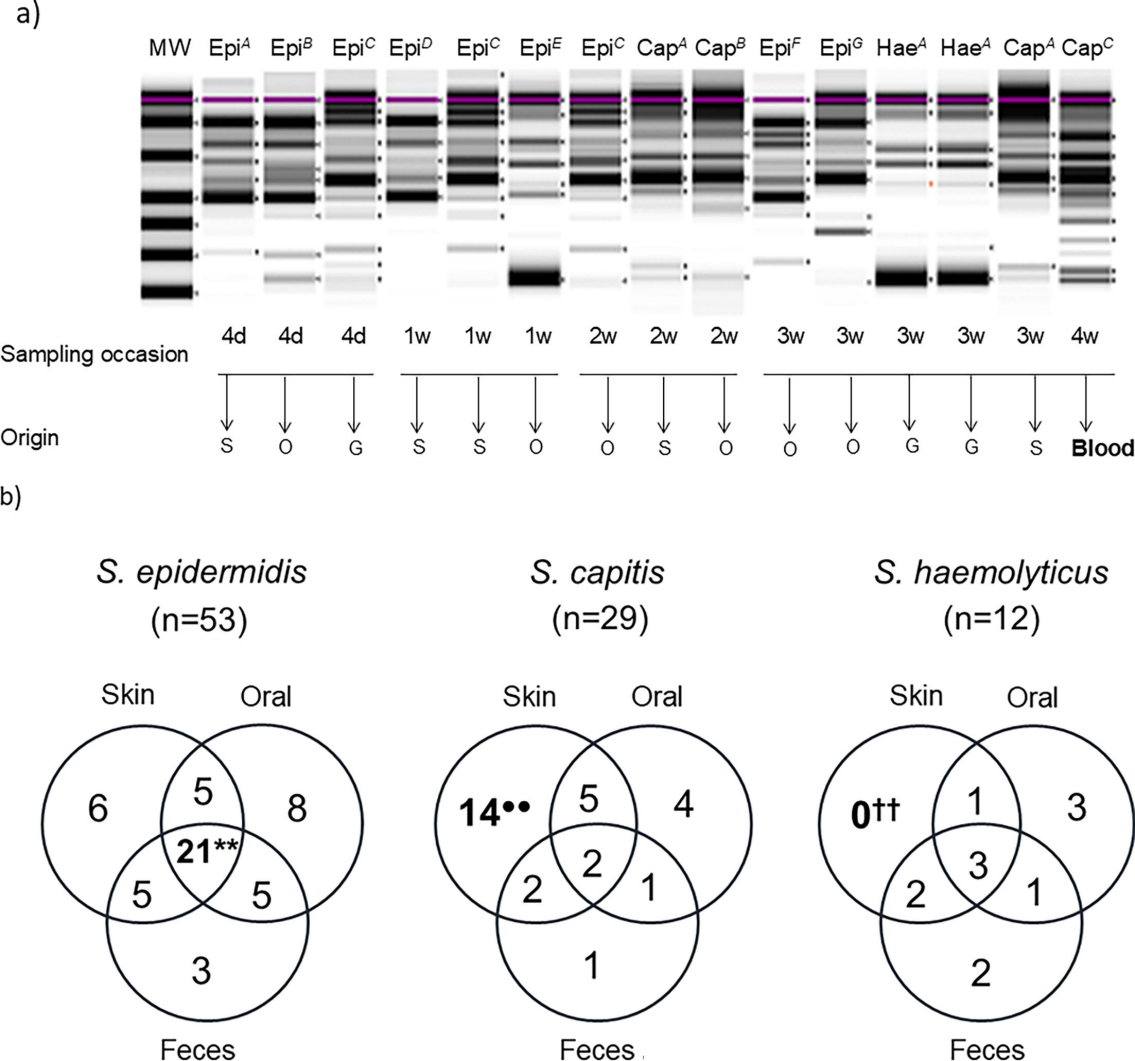
(a) RAPD patterns of all isolates of *S. epidermidis, S. capitis,* and *S. haemolyticus* obtained from one infant who developed septicemia at 4 weeks of age. Positive cultures were obtained at 4 days of age (4d) and at 1, 2, 3, and 4 weeks (**W**) of age, from the skin, (**S**), oral cavity (**O**), or gut (**G**), that is, from feces or rectal swabs. Isolates that show identical RAPD patterns are considered to belong to the same strain. Seven different *S. epidermidis* strains were identified (Epi^A^ - Epi^G^), two *S*. *capitis* strains were identified as colonizers, and Cap^A^ on the skin (2 and 3 weeks) and Cap^B^ in the mouth (2 w), whereas a third strain, Cap^C^, was detected in a blood culture. MW refers to the molecular weight marker. (b) Colonization of different body sites by individual *S. epidermidis, S. capitis,* and *S. haemolyticus* strains identified by RAPD. The strains derived from 12 infants who had a blood culture positive for CoNS. Proportions were compared using Fisher exact tests. A significantly higher proportion of *S. epidermidis* than *S. capitis* strains colonized all three body sites (*P* = 0.002). In addition, there was a significantly higher proportion of *S. epidermidis* strains than of *S. capitis* and *S. haemolyticus* strains together that colonized all sites (*P* = 0.005). ^●●^A significantly higher proportion of *S. capitis* than *S. epidermidis* strains colonized only the skin (*P* < 0.001). In addition, there was a significantly higher proportion of *S. capitis* than *S. epidermidis* and *S. haemolyticus* strains together that colonized the skin only (*P* = 0.0001). ††A significantly lower proportion of *S. haemolyticus* than *S. capitis* strains colonized only the skin (*P* = 0.003). In addition, there was a lower proportion of *S. haemolyticus* than *S. capitis* and *S. epidermidis* strains together that colonized the skin only (*P* = 0.06).

In order to determine whether the CoNS strains causing septicemia or contaminating blood cultures colonized one or several body sites of the affected infants prior to their isolation from the blood, we developed an RADP method for strain typing and optimized it to assess the strain identities of *S. epidermidis, S. capitis,* and *S. haemolyticus* isolates. This method readily identified different clones/strains within these species. Figure 3a shows the CoNS strains found in a single infant. One *S*. *capitis* strain (Cap^C^) was isolated from a blood culture drawn at infant age of 4 weeks. The RAPD pattern of this strain was clearly distinct from those of two other *S. capitis* strains, one of which was isolated from the skin (Cap^A^) at 2 and 3 weeks of age and one from the mouth (Cap^B^) at 2 weeks of age only (Fig. 3a). In addition, the infant carried seven *S*. *epidermidis* clones/strains (Epi^A^- Epi^G^) and a single *S. haemolyticus* strain (Hae^A^).

The strain typing method was applied to 16 CoNS blood isolates that were available for RAPD typing (two sepsis isolates and one isolate deemed as a blood culture contaminant were not stored at the clinical laboratory after species identification and could, therefore, not be analyzed further, see Table S3). In addition, all the CoNS isolates from the skin, oral cavity, and gut samples of the 12 infants yielding the blood isolates available for analysis were also analyzed by RAPD (318 *S*. *epidermidis,* 65 *S*. *capitis*, and 42 *S*. *haemolyticus* isolates). The RAPD patterns were not compared between infants.

Figure 3b shows the distributions at different body sites of the strains identified by RAPD. Of the 53 identified *S. epidermidis* strains, 21 (40%) were isolated from all three body sites (the skin, the oral cavity, and the gut) of the infant colonized. This was a significantly less-common pattern for the other two investigated CoNS species (40% vs 12%, *P* = 0.005). In contrast, *S. capitis* strains commonly colonized the skin only, a pattern that was significantly less-common among the other two investigated CoNS species (48% vs 9%, *P* = 0.0001). Indeed, none of the *S. haemolyticus* strains colonized the skin exclusively, a pattern that was unique to *S. haemolyticus* (0% vs 24%, *P* = 0.06).

### Tracking the origins of CoNS strains isolated from blood cultures

**Fig 4:**
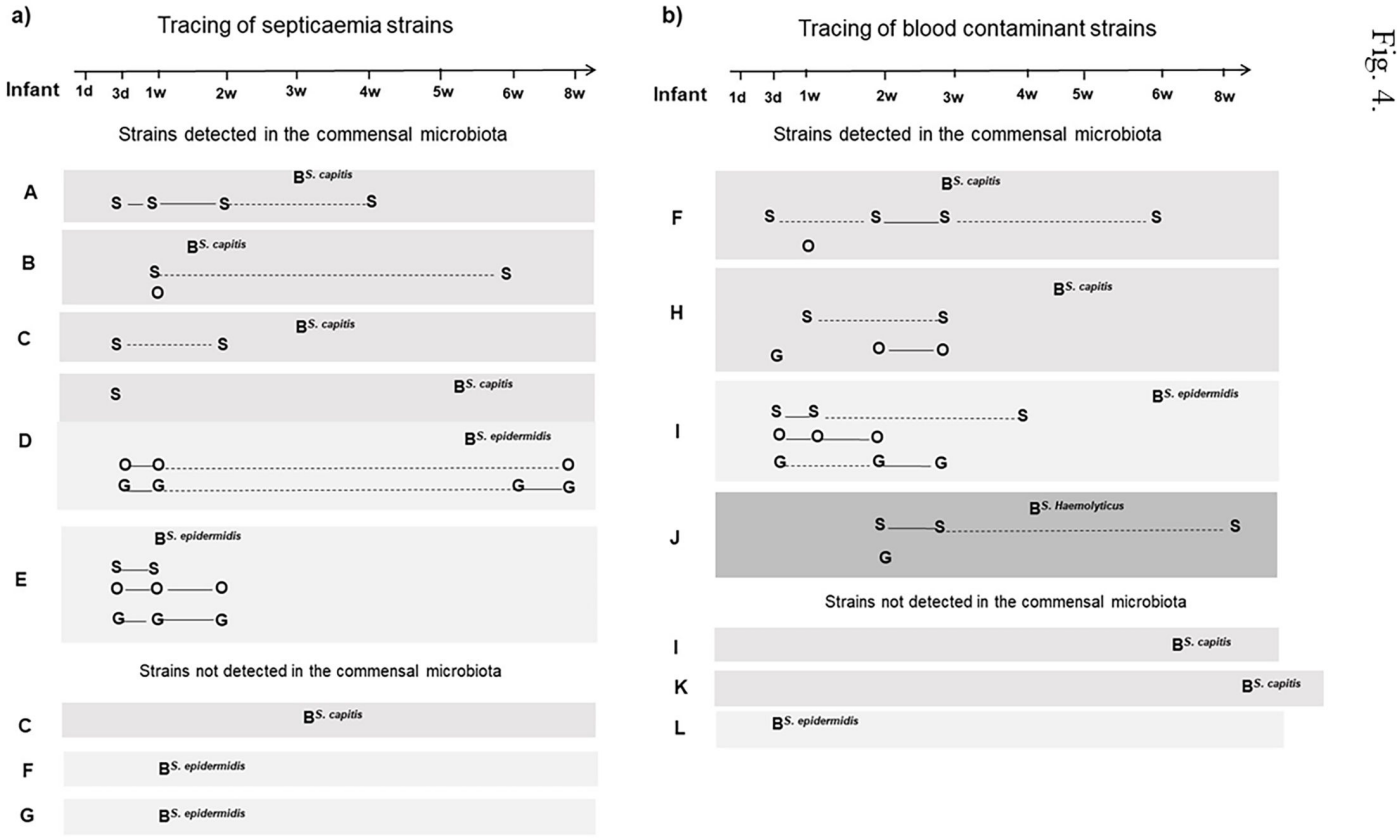
Identical CoNS strains in commensal microbiota and blood cultures from (a) episodes characterized as septicemia and (b) episodes not fulfilling the criteria for septicemia and yielding CoNS strains regarded as blood culture contaminants. Blood strains are indicated by **B,** and isolates resembling the blood strain but obtained from the skin (**S**), oral cavity (**O**), or gut (feces or rectal swabs) (**G**) are indicated. A solid line indicates the continuous presence of the blood CoNS strain at a particular body site, whereas a dashed line indicates that the strain was not detected on one or more intervening sampling occasions.

The RAPD pattern of each CoNS blood isolate was compared with the patterns of all commensal isolates of the same species that originated from the infant yielding the blood isolate. Isolates that showed RAPD patterns that were identical to those of the blood isolate were regarded as belonging to the same strain, which was thus deemed to have colonized the infant prior to its isolation from the blood. Figure 4 summarizes the results of the strain typing of CoNS isolates in 12 infants who had a CoNS in a blood culture. It shows that many of the strains isolated from blood could be traced back to the skin, oral cavity, or gut microbiota of the affected infant. Figure 4a shows the septicemic strains, and Fig. 4b shows the strains that were regarded as blood culture contaminants. Five infants (A–E) had CoNS septicemia caused by a strain that was also found in their commensal microbiota prior to the onset of septicemia (Fig. 4a). Infants A, B, and C had septicemia due to *S. capitis,* whereas infant D had a septic episode in which one *S*. *capitis* strain and one *S*. *epidermidis* strain were isolated from blood. Infant E had a septic episode that was due to an *S. epidermidis* strain. Two infants (F and G) had septic episodes that only involved CoNS strains that could not be identified as colonizers prior to the onset of septicemia (Fig. 4a). Note that infant C had two *S*. *capitis* strains in the blood during a septic episode, one of which was not found as a prior colonizer. In all, 6/9 septicemia strains were found to colonize the affected infant prior to the septic episode, including 4/5 of the *S. capitis* strains and 2/4 of the *S. epidermidis* strains. The *S. capitis* strains causing septicemia were found mostly on the skin or on the skin and in the oral cavity (infants A–D), whereas the *S. epidermidis* strains causing septicemia had a broader colonization pattern (infants D and E).

A similar pattern was seen for the blood strains that were regarded as blood culture contaminants (on clinical grounds) (Fig. 4b). In four cases (infants F, H, I, and J), *S. capitis* (two strains), *S. epidermidis,* and *S. haemolyticus* (one strain each) were identified as colonizers of the skin, the oral cavity, and/or the gut prior to their isolation from the blood. In three cases of blood contamination, two of which were caused by *S. capitis* and one by *S. epidermidis*, the blood strains could not be traced back to the commensal microbiota (infants I, K, and L).

In summary, in 10/16 cases (63%) with either CoNS septicemia or blood cultures contaminated with CoNS, the same CoNS strains were present in the commensal microbiota of the infants before they appeared in the bloodstream.

### Characteristics of the CoNS blood strains as revealed by whole genome sequencing

All blood strains available for analysis (*n* = 16) were subjected to whole genome sequencing. Eight of nine *S*. *capitis* strains, including the five *S*. *capitis* septicaemia strains and three of four strains regarded as blood culture contaminants, belonged to the NRSC-A clone and carried genes characteristic of this clone, including SCCmec, IS259, and the *icaADBC* operon. Five of these NRSC-A strains colonized the affected infant prior to its isolation from blood. The *S. capitis* blood strain, which did not belong to the NRCS-A clone, lacked the SCCmec and IS259 but harbored the *icaADBC* operon. This was a blood culture contaminant without any link to the infant’s microbiota.

The six *S*. *epidermidis* blood strains carried SCCmec, IS259, and the *icaADBC* operons. Three of six belonged to sequence type 2 (ST2), (one septicemia strain and two blood culture contaminants), and the other three strains (all septicemia strains) belonged to ST5, ST15, and ST59, respectively. Three of the six *S*. *epidermidis* strains (2 ST2 and 1 ST59 strain) colonized the affected infant prior to its isolation from blood.

The single *S. haemolyticus* strain belonged to ST1 and was a blood culture contaminant, which was also present in the microbiota of the affected infant.

### Putative virulence factors of the *S. capitis* and *S. epidermidis* strains

**Fig 5:**
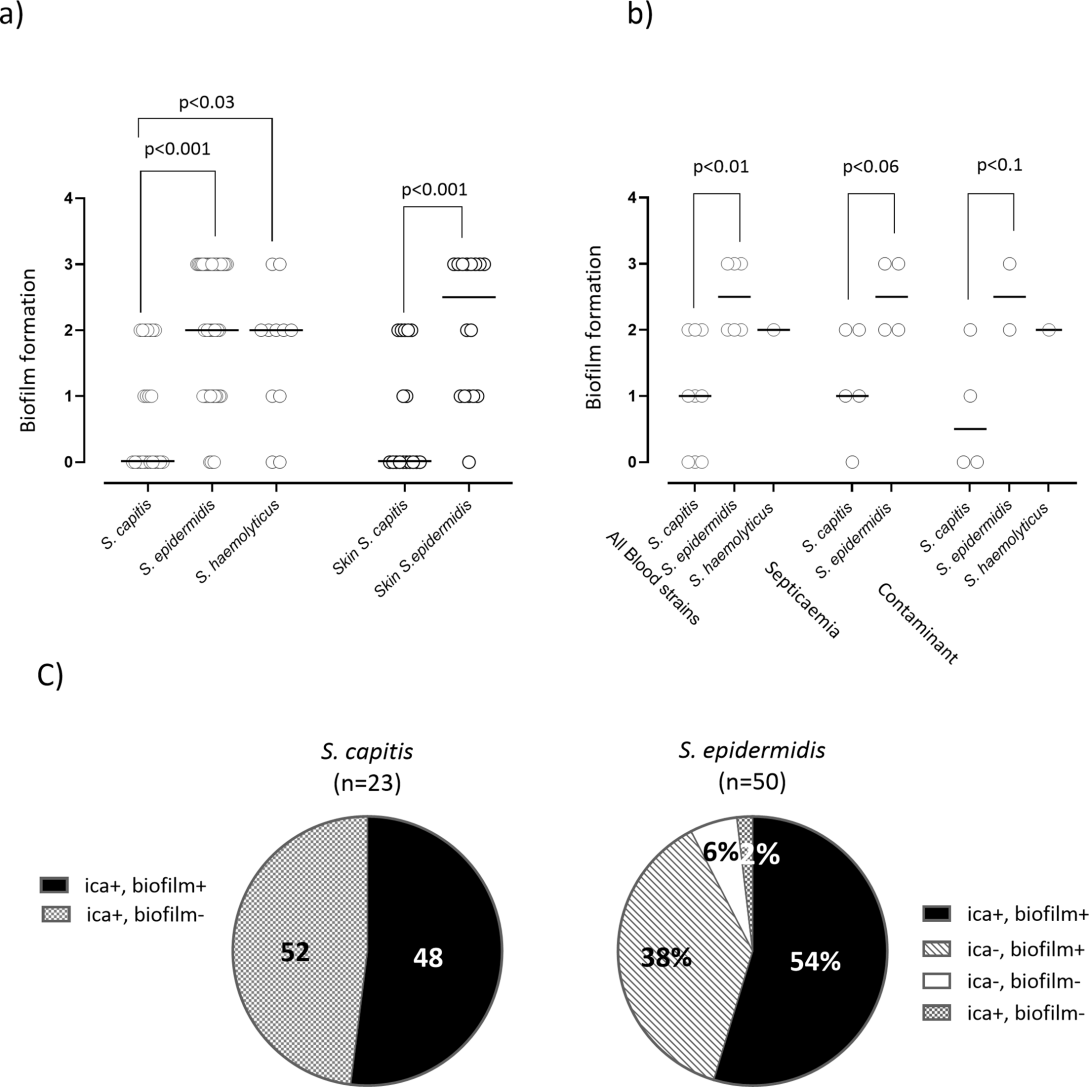
Biofilm formation by *S. epidermidis*, *S. capitis,* and *S. haemolyticus* strains was studied using a semi-quantitative method (15). All the strains (*n* = 94) identified in the skin, oral cavity, and rectal/fecal samples from 12 infants with at least one blood culture isolate available for analysis, as well as blood strains from these infants (*n* = 16) were assessed for biofilm formation. (a) The amounts of biofilm produced (graded 0, 1, 2, or 3) were compared between colonizing strains belonging to *S. epidermidis, S. capitis,* and *S. haemolyticus* within all the strains (*n* = 94), and within the strains obtained from the skin microbiota (*n* = 39) using Mann-Whitney exact *U*-tests. (b) Proportions of biofilm produced by CoNS were compared between *S. capitis* and *S. epidermidis* blood strains; *S. capitis* and *S. epidermidis* septicemia strains; and *S. capitis* and *S. epidermidis* blood strains regarded as blood culture contaminants (Mann-Whitney exact *U*-tests). (c) Biofilm production (yes or no) by colonizing *S. capitis* and *S. epidermidis* strains in relation to carriage of the *icaADBC* operon.

The capacity to produce a biofilm is recognized as a virulence trait of CoNS that cause infections *via* catheters or foreign materials (15). Here, all the blood strains from the 12 infants who had at least one CoNS blood isolate available for analysis, and all strains of *S. epidermidis*, *S. capitis* and *S. haemolyticus* colonizing these infants were screened for biofilm production *in vitro*. Among the colonizing strains, 92% of the *S. epidermidis* strains produced biofilm, compared with 52% of the *S. capitis* strains (*P* < 0.001). Using a semi-quantitative assessment and grading the biofilms on a scale of 0–3, it was found that the colonizing *S. epidermidis* and *S. haemolyticus* strains produced more biofilm than the corresponding *S. capitis* strains (median: 2 vs 0, *P* < 0.001 and 2 vs 0, *P* = 0.03; Mann-Whitney *U*-test) (Fig. 5a). Moreover, the *S. epidermidis* strains isolated from the skin microbiota produced significantly more biofilm than the skin-colonizing *S. capitis* strains (median: 2.5 vs 0, *P* < 0.001) (Fig. 5a), and this was also true for strains that exclusively colonized the skin (median: 3 vs 0, *P* = 0.005). The extent of biofilm formation did not differ significantly between the *S. capitis* and *S. epidermidis* strains isolated from the oral or gut microbiota (data not shown).

Among the strains isolated from the blood, the *S. epidermidis* strains produced significantly more biofilm than the *S. capitis* strains (median: 2.5 vs 1, *P* < 0.01) (Fig. 5b), and similar patterns were observed for both the strains that caused septicemia and the strains regarded as blood culture contaminants. We found no difference regarding biofilm formation between the blood strains and the colonizing strains of either *S. capitis* or *S. epidermidis,* but *S. epidermidis* produced more biofilm than *S. capitis*.

The strains tested for biofilm production were also tested by PCR for carriage of the *ica* operon, which encodes PIA, an essential factor for biofilm production. All the *S. capitis* and *S. epidermidis* strains isolated from the blood carried the *icaADBC* operon (Table 3). For *S. epidermidis*, carriage of the *ica* operon tended to be more frequent among the blood strains than among the strains only colonizing the infants (100% vs 56%, *P* = 0.07), whereas no difference was observed between the *S. capitis* blood and colonizing strains (100% vs 91%, *P* = 1.0). Among the commensal strains, the carriage rate of the *ica* operon was significantly higher among *S. capitis* strains than among *S. epidermidis* strains (91% vs 56%, *P* = 0.003, Fisher exact test) (Table 3).

**TABLE 3:** Carriage of putative virulence genes by *S. capitis* and *S. epidermidis* strains isolated from different body sites or from the blood samples of infants that yielded at least one blood culture that was positive for CoNS^*a*^

	Biofilm-associated genes			
	*S. capitis* (%)		*S. epidermidis*	
	No.	icaADBC	No.	icaADBC
Blood strains	9		6	
Septicemia	5	5 (100)	4	4 (100)
Blood culture contaminants	4	4 (100)	2	2 (100)
Colonizing strains	23	23 (100)	50	28 (56)

^*a*^ *S. capitis* (n=23) and *S. epidermidis* strains (n=50) colonizing the skin, oral cavity, and/or gut of infants who developed CoNS septicemia (N=7) and infants with no CoNS septicemia but with blood cultures that yielded CoNS regarded as blood culture contaminants (N=5), and all *S. epidermidis* (n=6) and *S. capitis* (n=9) blood strains were tested for the carriage of putative virulence factor-encoding genes using PCR. Carriage of the *icaADBC* genes (*ica* operon) was examined in both *S. capitis* and *S. epidermidis* strains*.*

Among the colonizing *S. epidermidis* strains, those carrying the *icaADBC* operon (*n* = 28) produced significantly more biofilm than strains lacking the operon (*n* = 22) (median: 3 vs 1, *P* = 0.003). This was true for *S. epidermidis* strains isolated from the gut (*n* = 20) (median: 3 vs 1, *P* = 0.007) and the oral cavity (*n* = 14) (median: 3 vs 1, *P* = 0.07), but not for skin-colonizing *S. epidermidis* strains (*n* = 16) (median: 3 vs 2, *P* = 0.8). In total, 92% of the colonizing *S. epidermidis* strains produced biofilms despite only 56% of them being *icaADBC-*positive. Only four *S*. *epidermidis* strains did not produce biofilms, three of which were negative and one of which was positive for the *icaADBC* operon (Fig. 5c).

Regarding the *S. capitis* strains colonizing the infants*,* all strains carried the *ica* operon, but only 48% of these produced a biofilm (Fig. 5c).

## DISCUSSION

In this study, we monitored the skin, oral, and gut colonization profiles of different CoNS species during the first 2 months of life in 42 extremely preterm infants, 9 of whom developed CoNS septicemia. We characterized the distributions of different CoNS species in the skin and oral and gut microbiota and optimized and applied RAPD for the differentiation of strains within the most common CoNS species. This enabled us to study the colonization patterns at the strain level and detect, in the microbiota, the CoNS strains that would subsequently cause septicemia. We also subjected all the CoNS blood isolates to whole genome sequencing to characterize these strains regarding clonal origin or sequence types (ST) and carriage of certain genetic determinants. Finally, we investigated the occurrence of putative virulence traits in CoNS strains that colonized and/or infected the infants.

The most prevalent CoNS species colonizing the infants was *S. epidermidis*, which represented more than two-thirds of all the commensal isolates, followed by *S. capitis* and *S. haemolyticus. S. capitis* showed a predilection for colonizing the skin, whereas most of the *S. epidermidis* strains colonized the skin, the gut, and the oral cavity. Colonization occurred very early, especially with *S. epidermidis* and particularly in the oral cavity, in that almost 40% of the infants harbored these bacteria already on the day of their birth. Colonization by *S. epidermidis* and *S. capitis* peaked during the first weeks of life and thereafter declined quite rapidly. This likely reflects increased competition from other bacteria that are establishing themselves in the microbiota (44) and/or increased production of defense molecules, such as antimicrobial peptides.

Nine infants developed CoNS-related septicemia. We found *S. capitis* to be the major cause, followed by *S. epidermidis*. Thus, *S. capitis* contributed 7/11 septicemia isolates (64%) but represented only 18% of the commensal CoNS isolates from the infants who developed septicemia (*P* = 0.001). Furthermore, these infants had a significantly higher proportion of *S. capitis* in their CoNS microbiota, and most notably, significantly increased oral colonization in the first 2 weeks of life. Thus, commensal colonization by *S. capitis,* especially colonization of the oral cavity, was a significant risk factor for the development of CoNS septicemia in these extremely preterm infants.

Despite this finding, the *S. capitis* septicemia strains could not be traced primarily to the oral microbiota, although they were in most cases identified as colonizers of the infant’s skin prior to appearing in the bloodstream. The reason for this discrepancy is unclear. Colonization of the oral cavity by *S. capitis* might reflect impaired host defenses, as *S. capitis* seems most naturally to be fit for skin colonization, and this impairment could also predispose the host to an inability to keep the bloodstream sterile. Nevertheless, direct translocation across the oral mucosa should not be excluded, as it readily occurs at least in an animal model (13). If translocation follows rapidly after establishment and proliferation of the strain in the oral mucosa, oral samples obtained prior to sepsis development may well be negative.

Interestingly, all *S. capitis* septicaemia strains and most *S. capitis* blood strains regarded as blood culture contaminants belonged to the NRCS-A clone, a globally spread *S. capitis* clone recognized as an increasingly important cause of septicaemia in preterm infants (45). In another Swedish study, more than two-thirds of the *S. capitis* blood isolates from NICU patients were found to be related to this clone (46).

The NRCS-A clone is multiresistant and shows variable sensitivity to vancomycin, and Butin et al. reported NRCS-A as a primary cause of late-onset sepsis especially in vancomycin-treated preterm neonates (27) and also suggested that disruption of the gut microbiota by vancomycin treatment may increase the risk of translocation of this strain across the mucosa, resulting in septicemia (27, 47). However, they did not investigate oral colonization by *S. capitis* or the NRCS-A clone specifically. Other studies also suggest that the port of entry of NRCS-A into the bloodstream may be mucosal sites rather than the skin and indwelling catheters. In the study by Wang and coworkers, no cases of catheter-related *S. capitis* septicaemia were identified (48). Also, the poor or absence of biofilm production in most *S. capitis* isolates argues against catheter-related infections (49). A case report described a persistent septicaemia episode caused by *S. capitis* in a neonate who did not have any central catheter at the time of the sepsis, and the strain was suggested to originate in the gut (50). Gut colonization by *S. capitis* in preterm neonates is not uncommon (27). To our knowledge, our study is the first to describe the oral *S. capitis* colonization pattern in preterm neonates.

In the present study, treatment with meropenem seemed to promote oral colonization by *S. capitis*, whereas we observed no effect of previous vancomycin treatment on *S. capitis* colonization. However, both meropenem and vancomycin treatment seemed to suppress gut colonization by *S. epidermidis*.

*S. epidermidis* was the second most common cause of CoNS septicemia. Others have found *S. epidermidis* to be the most common cause of septicemia in very-low-birth infants (7, 51), followed by *S. capitis* (7, 48) or *S. haemolyticus* (51).

The *S. epidermidis* strains showed broad colonizing abilities and were the most commonly detected species at all sampling sites throughout the first 2 months of life. At the strain level, the most common pattern was that the same strain appeared on the skin, in the mouth, and in the feces of an infant. *S. epidermidis* strains causing septicemia could be found in the oral cavity, in the gut, as well as on the skin prior to its isolation from the bloodstream, making it difficult to determine the port of entry. Previous observations have suggested that mucosal sites are important sources of *S. epidermidis* strains that are later found in the blood (52, 53).

Bradford et al., who investigated CoNS in very low-birth weight infants, suggested that blood culture contaminants and septicemia isolates derive from the same pool of hospital strains, which are both successful colonizers and capable of causing sepsis in neonates (7). We did not examine the molecular relatedness of the strains from different infants; hence, we can not determine whether or not the infants examined here were colonized by hospital strains spread in the NICU. However, five of the six *S*. *epidermidis* strains isolated from blood cultures, of which half also colonized the affected infants, belonged to sequence types ST2, ST5, and ST59, which commonly represent nosocomial strains with pathogenic potential (25).

The virulence factors of CoNS strains that cause septicemia have not been fully explored. We assessed all the strains of *S. capitis*, *S. epidermidis,* and *S. haemolyticus* from infants with blood cultures that were positive for CoNS, for the carriage of putative virulence genes, and for biofilm production. Almost all the *S. capitis* strains, irrespective of whether they were only colonizing an infant or isolated from blood, carried the *ica* operon, which is in accordance with the findings of other studies (20, 49). For *S. epidermidis*, the strains isolated from the bloodstream tended to carry the *ica* operon more frequently that the commensal *S. epidermidis* strains from the same infants. All of the blood strains, but only 56% of the commensal *S. epidermidis* strains carried the *ica* operon (*P* = 0.07), suggesting a role for these genes in septicemia development. Enrichment of the *ica* operon among *S. epidermidis* septicemia isolates was also observed by Frebourg and coworkers (54), although subsequent studies did not confirm these findings (19, 21, 32).

The vast majority of the *S. epidermidis* strains (92%) were biofilm producers. This rate is considerably higher than the rates reported in previous studies (22%–63%) using the same methodology (15, 55). Variations of the proportions of *S. epidermidis* strains that produce biofilms are to be expected, since biofilm production by individual *S. epidermidis* strains might be highly variable and greatly influenced by environmental and growth conditions (32). Phase variation in *S. epidermidis* biofilm production has been suggested to contribute to virulence by promoting the detachment and dissemination of planktonic cells from the mature biofilm (56).

Within the *S. epidermidis* strains, we did not find any differences in the amounts of biofilm produced between septicemia strains and strains confined to the skin or mucous membranes or between septicemia strains and strains identified as blood culture contaminants. In agreement with this, de Silva et al. (21) found no significant differences in biofilm production between *S. epidermidis* skin strains from healthy infants and CoNS bacteremia cases, or between *S. epidermidis* bacteremia blood strains and strains regarded as blood culture contaminants when using the qualitative Congo Red agar method. However, when biofilm production was measured quantitatively in microtiter plates, the levels were significantly higher in both skin and blood *S. epidermidis* strains obtained from infants with *S. epidermidis* septicemia, compared with *S. epidermidis* strains from healthy controls and CoNS blood culture contaminants, respectively (21). This indicates that the regulation of biofilm expression plays a central role in the disease process, although we were unable to detect such differences in the amounts of biofilm produced between commensal and septicemia strains in our relatively small study.

Biofilm production was also observed for the *S. epidermidis* strains that lacked the *icaADBC* operon, which is in accordance with studies of strains causing infections of the joints (19). However, the *ica*-positive *S. epidermidis* strains produced larger amounts of biofilm than the *ica*-negative strains, which is also in agreement with the results of a previous study (19).

In contrast, all the blood strains and all colonizing strains of *S. capitis* carried the *ica* operon, although only approximately half of these strains produced biofilms *in vitro*. Accordingly, the majority of the *S. capitis* strains from bloodstream infections in neonates (49) and persistent, prosthetic device-related infections (57) have been shown not to produce biofilms. The *S. capitis* strains produced significantly less biofilm than the *S. epidermidis* strains, despite a higher carriage rate of the *ica* operon, which is also in agreement with previous studies (58). This may suggest that the *in vitro* conditions applied in the current and other studies are not optimal for *S. capitis* biofilm production, perhaps because they poorly reflect the prevailing conditions on the skin, which appears to be the preferred niche for *S. capitis*. Alternatively, the *ica* operon may primarily serve purposes other than biofilm production in this species. We did not find any *icaADBC-*negative *S. capitis* strains that produced biofilm, in agreement with previous observations (21).

*S. capitis* can be further divided into the *capitis* and *urealyticus* sub-species, with the latter being reported more often to produce biofilm (20). However, since we did not type our *S. capitis* strains to the sup-species level, we were unable to investigate this issue. For both *S. capitis* and *S. epidermidis*, we found no relationship between invasiveness and biofilm production, which suggests that the importance of biofilm formation is not greater for spread to and survival in the bloodstream than it is for skin or mucosal colonization.

In conclusion, extremely preterm infants are colonized by CoNS on the skin and in the mouth and gut at a very early stage in life. *S. epidermidis* strains dominate at all the body sites, although *S. capitis* is also commonly detected, especially on the skin, and this species, or more specifically the *S. capitis* NRCS-A clone, was the most common cause of CoNS septicemia. *S. capitis* was enriched among blood strains compared with commensal strains, and early colonization of the oral cavity by *S. capitis* was associated with an increased risk of CoNS septicemia. However, most of the *S. capitis* strains isolated from blood cultures were primarily traced back to the skin microbiota. *S. epidermidis* strains usually exhibited a broad colonization pattern, and their preferred site of entry into the bloodstream could not be determined. Biofilm production in both *S. epidermidis* and *S. capitis* could not be linked to invasive capacity.

## SUPPLEMENTAL MATERIAL

Fig. S1 aem.00980-24-s0001.tif Colonization by different CoNS species in relation to various perinatal factors.

Fig. S2 aem.00980-24-s0002.tif Colonization by different CoNS species in relation to treatment by certain antibiotics.

Supplemental legends aem.00980-24-s0003.docx Legends for Fig. S1 and S2.

Supplemental tables aem.00980-24-s0004.docx Tables S1 to S4.

ASM does not own the copyrights to Supplemental Material that may be linked to, or accessed through, an article. The authors have granted ASM a non-exclusive, world-wide license to publish the Supplemental Material files. Please contact the corresponding author directly for reuse.
