# Supplementary figures and images for: Tracing Staphylococcus capitis and Staphylococcus epidermidis strains causing septicemia in extremely preterm infants to the skin, mouth, and gut microbiota
Source: Appl Environ Microbiol. 2024 Dec 18;91(1):e00980-24. doi: 10.1128/aem.00980-24 (PMC11784025; doi:10.1128/aem.00980-24)

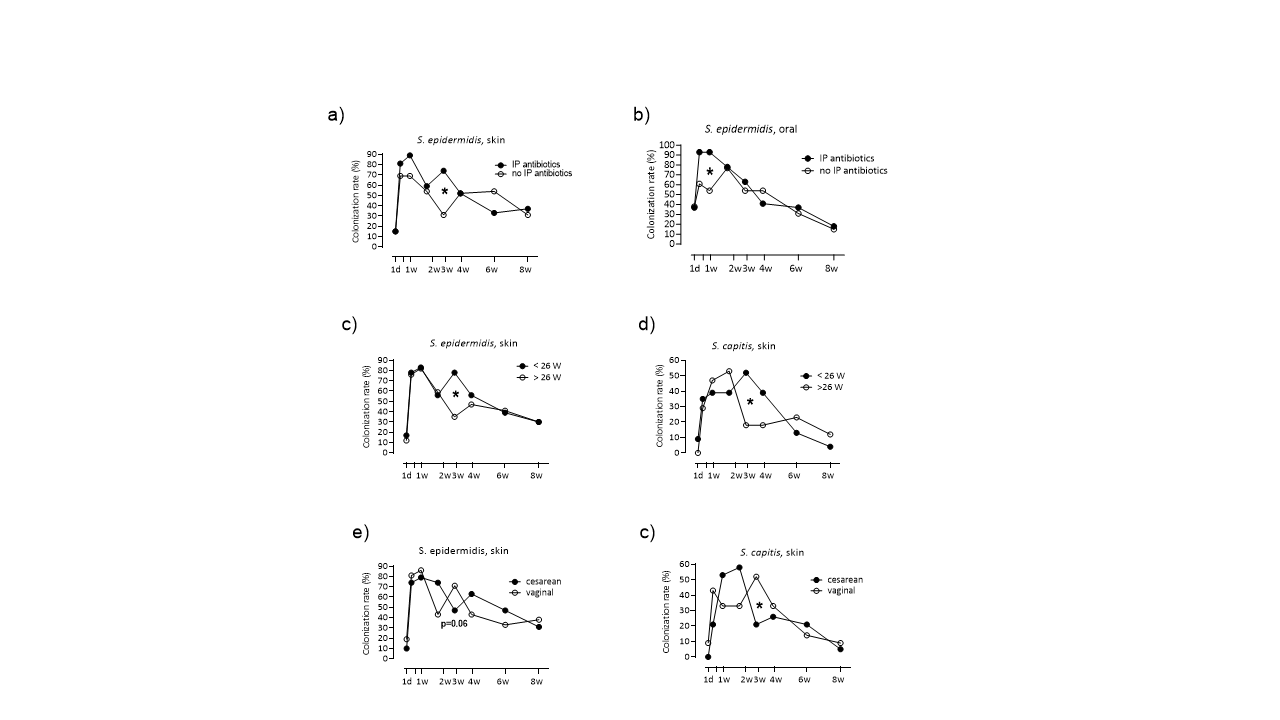

Supplement: Fig. S1 — Colonization by different CoNS species in relation to various perinatal factors. [file aem.00980-24-s0001.tif]

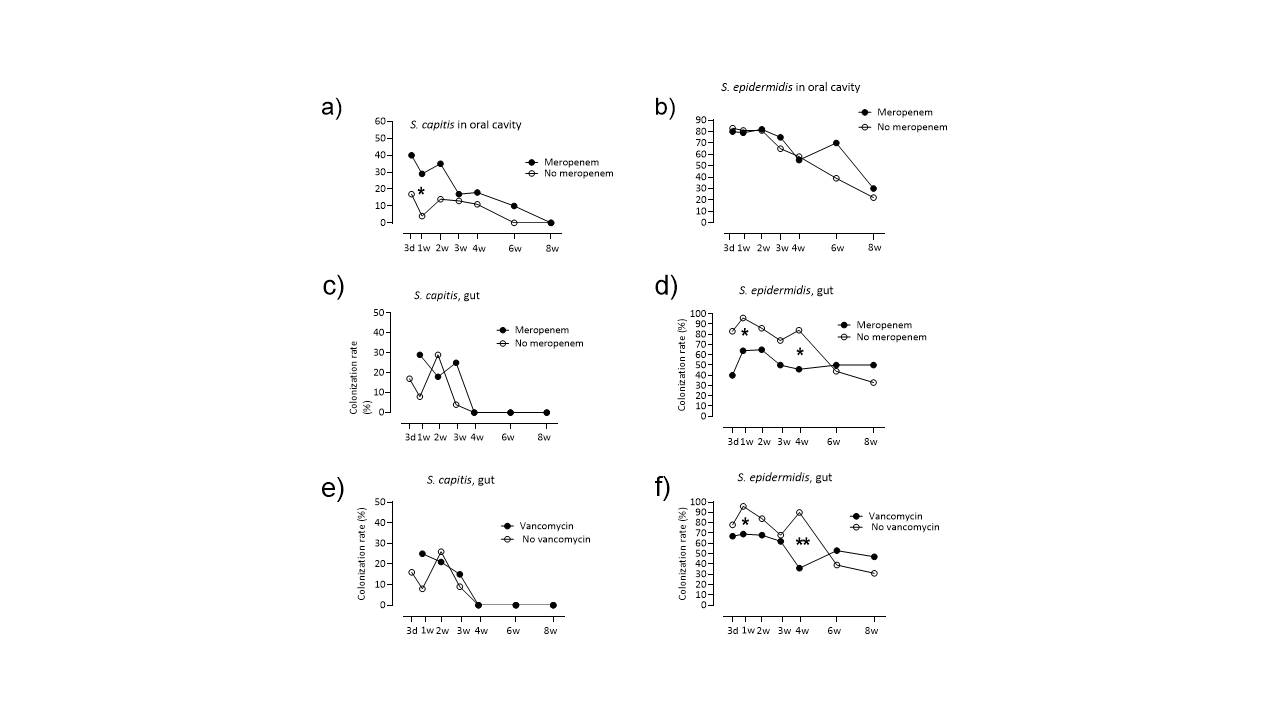

Supplement: Fig. S2 — Colonization by different CoNS species in relation to treatment by certain antibiotics. [file aem.00980-24-s0002.tif]
