# Supplementary material for: Tracing Staphylococcus capitis and Staphylococcus epidermidis strains causing septicemia in extremely preterm infants to the skin, mouth, and gut microbiota
Source: Appl Environ Microbiol. 2024 Dec 18;91(1):e00980-24. doi: 10.1128/aem.00980-24 (PMC11784025; doi:10.1128/aem.00980-24)
Supplement: Supplemental legends — Legends for Fig. S1 and S2. [file aem.00980-24-s0003.docx]

Figure legends:

**Fig. 1.** Colonization by different CoNS species on the skin, in the oral cavity or in the gut of infants in relation to various perinatal factors. Colonization of *S. epidermidis* **a**) on the skin and **b**) in the oral cavity of infants who were exposed and infants who were not exposed to antibiotics during partus. Colonization of the skin by **c**) *S. epidermidis* and **d**) *S. capitis* in infants born at <26 weeks or ≥26 weeks of gestation. Colonization of the skin by **e**) *S. epidermidis* and **f**) *S. capitis* in infants who were delivered *via* the vaginal route or by Cesarean section.

**Fig. 2.** Colonization by different CoNS species of the skin, the oral cavity or the gut of infants who were exposed and of infants who were not exposed to certain antibiotics (meropenem, cloxacillin or vancomycin) during the first 8 weeks of life. (**a-b**) Potential impacts of meropenem on oral colonization by *S. capitis* or *S. epidermidis*. (**c-d**) Potential impact of meropenem on gut colonization by *S. capitis* or *S. epidermidis*. (**e-f**) Potential impact of vancomycin on gut colonization by *S. capitis* or *S. epidermidis.*
