# Supplementary material for: Tracing Staphylococcus capitis and Staphylococcus epidermidis strains causing septicemia in extremely preterm infants to the skin, mouth, and gut microbiota
Source: Appl Environ Microbiol. 2024 Dec 18;91(1):e00980-24. doi: 10.1128/aem.00980-24 (PMC11784025; doi:10.1128/aem.00980-24)
Supplement: Supplemental tables — Tables S1 to S4. [file aem.00980-24-s0004.docx]

Supplementary Tables:

Table 1. Antibiotics administered to the infants included in the current study.

| **No. (%) of infants receiving antibiotics at age** | | | | | | |  |
| --- | --- | --- | --- | --- | --- | --- | --- |
|  | 0–3 days | 4–7 days | 2 weeks | 3 weeks | 4 weeks | 5–6 weeks | |
| Penicillin G | 42 (100) | 34 (85) | 8 (21) | 0 (0) | 0 (0) | 1 (4) | |
| Tobramycin | 42 (100) | 35 (88) | 16 (42) | 6 (17) | 4 (13) | 9 (32) | |
| Cloxacillin | 0 (0) | 10 (25) | 26 (68) | 16 (46) | 9 (30) | 11 (39) | |
| Vancomycin | 3 (7) | 16 (40) | 19 (50) | 13 (37) | 11 (37) | 15 (54) | |
| Meropenem | 6 (14) | 14 (35) | 17 (45) | 12 (34) | 11 (37) | 10 (36) | |
| Other^a^ | 2 (5) | 2 (5) | 2 (5) | 2 (6) | 1 (3) | 5 (18) | |
| **No. infants**  **in the ward** | 42 | 40 | 38 | 35 | 30 | 28 | |

The table shows the frequency of infants treated with different antibiotics (prophylactic or therapeutic) at various timepoints after birth in the studied sub-cohort (N=42). At the time of the study, prophylactic treatment with penicillin G and tobramycin, often followed by cloxacillin, was provided to all extremely preterm neonates in the NICU for as long as umbilical lines were in place.

**^a^**Trimethoprim, Cefotaxime, Cefuroxime, Doctacillin, Ampicillin.

Table 2. Antibiotic susceptibility patterns of the blood culture CoNS isolates

| % of resistant CoNS strains | | | | | | | | |
| --- | --- | --- | --- | --- | --- | --- | --- | --- |
| CoNS species | Isoxapenicillin | Meropenem | | Cefotaxim | Tobramycin | Clindamycin | Fusidic acid | Vancomycin |
| *S. capitis* (11) | 90% | 90% | 90% | | 90% | 20% | 80% | 0% |
| *S. epidermidis* (7) | 100% | 100% | 100% | | 100% | 57% | 43% | 0% |
| *S. haemolyticus* (1) | 100% | 100% | 100% | | 100% | 100% | 100% | 0% |

The table shows the antibiotic susceptibility testing that was performed at the Clinical Microbiology laboratory at Sahlgrenska University Hospital as part of routine blood culture analysis,

Table 3. *S. epidermidis, S. capitis* and *S. haemolyticus* isolates detected in blood cultures from infants with and without CoNS septicemia.

Of 19 CoNS blood isolates originating from 14 of the study infants, 16 were available for RAPD strain typing and additional analyses: 9 isolates (5 *S. capitis,* 4 *S. epidermidis*) from 7 infants with CoNS septicemia and 7 isolates (4 *S. capitis*, 2 *S. epidermidis* and 1 *S. haemolyticus*) from 6 infants with CoNS as blood culture contaminants.

*Three blood culture isolates were lost to analysis because they were not saved after isolation at the clinical microbiology laboratory: two *S. capitis* septicemia isolates and one *S. epidermidis* isolate (regarded as a blood culture contaminant) from an infant without CoNS septicemia.

**Two of these infants also had CoNS septicemic episodes.

Table 4. The accession numbers of genome sequences of the CoNS blood isolate

| Species | Strain | Accession number |
| --- | --- | --- |
| Staphylococcus epidermidis | 87B20656b | JBJGEU000000000 |
| Staphylococcus capitis | 81B23416 | JBJGEV000000000 |
| Staphylococcus capitis | 71B2147b | JBJGEW000000000 |
| Staphylococcus capitis | 71B2147a | JBJGEX000000000 |
| Staphylococcus epidermidis | 61B38366 | JBJGEY000000000 |
| Staphylococcus capitis | 61B38254 | JBJGEZ000000000 |
| Staphylococcus capitis | 59B36916 | JBJGFA000000000 |
| Staphylococcus epidermidis | 59B36734 | JBJGFB000000000 |
| Staphylococcus capitis | 55B31579 | JBJGFC000000000 |
| Staphylococcus epidermidis | 55B30108 | JBJGFD000000000 |
| Staphylococcus haemolytic | 51B19177 | JBJGFE000000000 |
| Staphylococcus epidermidis | 49B13437 | JBJGFF000000000 |
| Staphylococcus epidermidis | 49B13047 | JBJGFG000000000 |
| Staphylococcus capitis | 15B27408 | JBJGFI000000000 |
| Staphylococcus capitis | 88B23939 | JBJGFJ000000000 |
| Staphylococcus capitis | 4B14864 | JBJKFF000000000 |

The whole-*genome sequences* of the CoNS blood isolates were submitted to the

NCBA database. Thus, genome sequences are available under the accession numbers

included in the supplementary Table 4.
